# Phospholipid Composition and Electric Charge in Healthy and Cancerous Parts of Human Kidneys

**DOI:** 10.1007/s00232-013-9554-7

**Published:** 2013-05-07

**Authors:** Barbara Szachowicz-Petelska, Izabela Dobrzyńska, Marta Skrodzka, Barbara Darewicz, Zbigniew A. Figaszewski, Jacek Kudelski

**Affiliations:** 1 Institute of Chemistry, University in Białystok, Al. Piłsudskiego 11/4, 15-443 Białystok, Poland; 2 Clinical Department of Urology, Medical University of Białystok, Skłodowskiej-Curie 24A, 15-276 Białystok, Poland; 3 Laboratory of Electrochemical Power Sources, Faculty of Chemistry, University of Warsaw, Pasteur St. 1, 02-093 Warsaw, Poland

**Keywords:** Electric charge, Phosphatidylcholine, Phosphatidylethanolamine, Phosphatidylinositol, Phosphatidylserine, Renal cancer

## Abstract

Phospholipids are ubiquitous in nature and are essential for the lipid bilayer of cell membranes. Their structural and functional properties are pivotal for the survival of the cell. In this study the phospholipids of healthy and cancerous human renal tissues from the same patients are compared with special reference to the electric charge of the membrane. A simple and highly effective normal-phase method is described for analyzing phospholipids content. This work is focused on changes of phospholipids content (PtdIns, phosphatidylinositol; PtdSer, phosphatidylserine; PtdEtn, phosphatidylethanoloamine; PtdCho, phosphatidylcholine) in cell membranes of renal cancer of pT1 stage, G2 grade, without metastasis. Surface charge density of healthy and cancerous human renal tissues was measured by electrophoresis. The measurements were carried out at various pH of solution. Depending on the surface charge density as a function of pH, acidic (*C*_TA_) and basic (*C*_TB_) functional group concentrations and their average association constants with hydrogen (*K*_AH_) or hydroxyl (*K*_BOH_) ions were evaluated. The process of cancer transformation was accompanied by an increase in total amount of phospholipids as well as an increase in *C*_TA_ and *K*_BOH_, whereas *K*_AH_ and *C*_TB_ were decreased compared with unchanged tumor cells.

## Introduction

Cancer of the kidney accounts for approximately 2–3 % of all cancers, with the highest incidence in the more developed countries. Renal cell carcinomas (RCC) comprise nearly 85 % of kidney cancers. This cancer occurs mainly in patients between the ages of 50 and 70 years, and men are affected twice as often as women (Hoffmann et al. 2005). The etiology of RCC is unknown, although epidemiological studies have suggested a relationship between the habit of smoking and RCC. Furthermore, many dietary factors have been considered in relation to RCC. The observed relationship between the diet and cancer has raised interest in protective and physiologically active food ingredients (Hemminki et al. 2002).

Tumor cells produce and excrete to blood numerous substances that are present in the cell itself in trace amounts only. They are called tumor biomarkers. Their physicochemical properties are diverse and they are classed to several groups: cancer-fetal antigens, carcinogenic antigens, proteins, cell metabolism product, cell growth factors, hormones, enzymes and isoenzymes (Szachowicz-Petelska et al. 2002). They are transported to the blood circulation system across the cell membrane. Therefore, the estimation of the state of the tumor cell membrane is likely to be essentially that in the studies of tumor biology.

The most important properties of a biological membrane are its electric charge and its potential drop between the membrane and surrounding solution. Electric properties of the membrane are determined by acid–base and complex formation equilibria of membrane and solution components (Szachowicz-Petelska et al. 2012). As a main component of cell membrane, phospholipids are involved in those equilibria.

Many discoveries have shown that phospholipids are not limited only to being structural components of the cell membrane. Phospholipids are vital in some essential biological functions in live organisms. They are important in maintaining cell membranes integrity and play a significant role in functions such as: membrane permeability, membrane fluidity, membrane interactions (lipid–protein) and membrane deterioration (Bezrukov 2000; White et al. 2001). Phospholipids are involved in metabolism-related and neurological diseases (Lohmeyer and Bittmann 1994; Lee 1998) and in the regulation of basic biological processes as signaling compounds (Izumi and Shimizu 1995; Hannun et al. 2001).

The examining of electric charge may reveal a lot of information about the membrane and about the balance between components of membrane, but also between components of membrane and the components of solution. The work is focused on changes of phospholipids content and electric charge that occur in cell membranes of renal cancer.

## Materials and Methods

The study project has been approved by local research ethics committee of Medical University of Bialystok. Tissue samples were obtained from 9 patients (4 men and 5 women) who underwent nephrectomy because of renal cancer. Tumor staging system consists of 4 TNM stages—lower number is related to better clinical prognosis. Our study included only low stage renal cancers classified histopathologically as renal clear cell cancer in G2 grade in Fuhrman scale and pT1 stage. None of the patients had lymph nodes (N0) involved at the time of diagnosis. The age of patients ranged from 37 to 74 years old. Tumor samples with healthy renal tissue were collected immediately after tumor removal.

### Isolation of Cell Membrane

The tissues (about 1 g) were homogenized in 1 mM NaHCO_3_ (pH 7.6), 0.5 mM CaCl_2_, in a loose-fitting Dounce homogenizer. Membrane fragments were separated from nuclei and mitochondria by rate-zonal centrifugation of the low-speed pellet as described by Evans (1970). The sediment was homogenized in sucrose (1.22 g/cm^3^ density). Next, the sediment was covered with sucrose (1.16 g/cm^3^ density) and the cell membranes were separated by centrifugation at 2,000 × *g* for 25–35 min (Dobrzyńska et al. 2005).

### Isolation and Analysis of Phospholipids by HPLC Method

The method of Folch (1957) was used to extract phospholipids. The cell membrane was homogenized in a chloroform–methanol mixture of (2:1 volume ratio). The solution was then filtered with degreased paper filters, and the precipitate was washed with an extracting solution (8:4:3 chloroform:methanol:aqueous calcium chloride solution 0.05 M). The suspension was centrifuged at 500 × *g* for 2 min, the organic and the aqueous phases were separated, and the aqueous phase was shaken again with chloroform, methanol and water mixture of (3:48:47 volume ratio) and the phases were separated. The organic phases were combined and evaporated to dryness. The extract was dissolved in 200 μl of hexane:isopropanol mixture (3:2) (Dobrzyńska et al. 2005). Addition of 0.03 % *tert*-butylhydroxytoluene (BHT) and flushing with nitrogen at each step in the procedure were used to prevent oxidation during lipid extraction.

HPLC analysis was performed on the extracted phospholipids to assess the quantities phosphatidylinositol (PtdIns), phosphatidylserine (PtdSer), phosphatidylethanolamine (PtdEtn) and phosphatidylcholine (PtdChol). The isolated phospholipids were separated by group analysis in a silica gel column using normal phase (NP) HPLC; acetonitryle–methanol–phosphoric acid (85 %) mixture (130:5:1,5 volume ratio) by isocratic elution at 1 ml/s flow rate and 214 nm wavelength (Dobrzyńska et al. 2005).

### Electrochemical Method

In order to determine surface charge density of cell membrane, renal cancer tissue from human was exposed to trypsin action. Received cells were suspended in 0.015 M NaCl and put into the measuring vessel; then electrophoretic mobility was measured by the Zetasizer Nano ZS apparatus (Malvern Instruments). The measurements were carried out as a function of pH.

The surface charge density has been determined by the equation $\delta = \frac{\eta \cdot u}{d}$; here, *u* indicates electrophoretic mobility, *η*, viscosity of solution, and *d*, diffuse layer thickness (Krysiński and Tien 1986). The diffuse layer thickness was determined from the formula $d = \sqrt {\frac{{\varepsilon \cdot \varepsilon_{0} \cdot R \cdot T}}{{2 \cdot F^{2} \cdot I}}}$ (Barrow 1996), where *R* is the gas constant, *T* is the temperature, *F* is the Faraday number, *I* is the ionic strength of 0.9 % NaCl, and *εε*_o_ are the relative and absolute permittivities of the medium.

Acid (*C*_TA_) and basic (*C*_TB_) functional group concentrations and their average association constants with hydrogen (*K*_AH_) or hydroxyl (*K*_BOH_) ions were determined as described previously (Dobrzyńska et al. 2006).

### Statistical Methods

The data obtained in this study are expressed as mean ± SD. The data were analyzed by the Wilcoxon matched pairs signed ranks test using SPSS software, version 8.0, for comparisons between control and cancer samples. Values of *p* of <0.05 were considered significant.

## Results

**Fig. 1:**
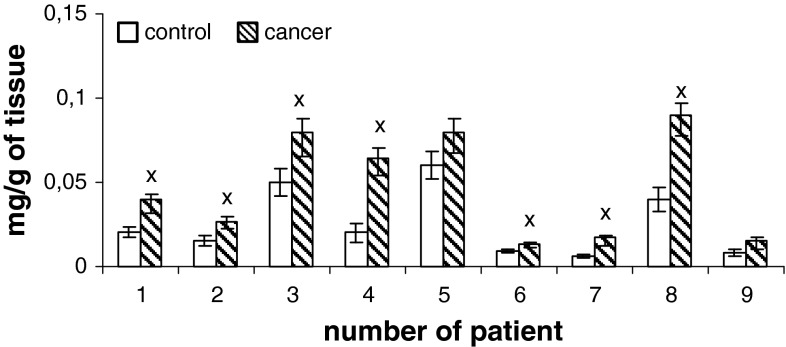
Content of phosphatidylinositol of cell membranes in human renal cancer of pT1 stage, G2 grade, without metastasis. Statistically significant differences for *p* < 0.05. ^x^ In comparison with control

**Fig. 2:**
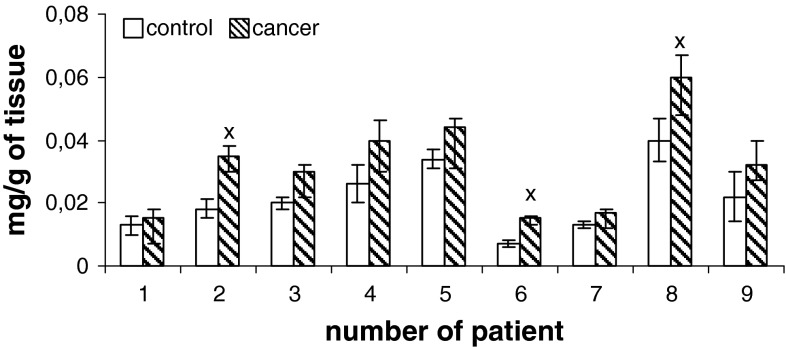
Content of phosphatidylserine of cell membranes in human renal cancer of pT1 stage, G2 grade, without metastasis. Statistically significant differences for *p* < 0.05. ^x^ In comparison with control

**Fig. 3:**
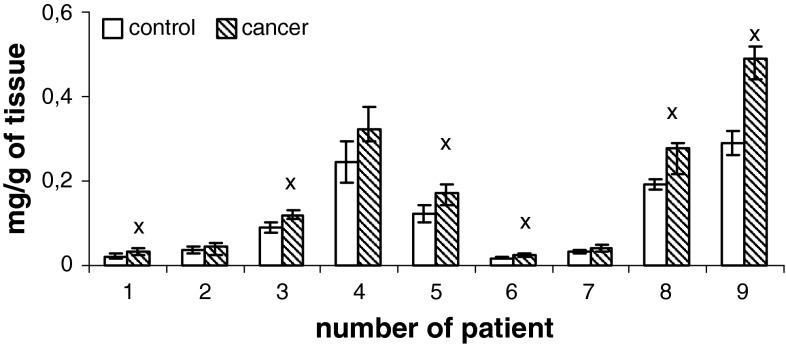
Content of phosphatidylethanolamine of cell membranes in human renal cancer of pT1 stage, G2 grade, without metastasis. Statistically significant differences for *p* < 0.05. ^x^ In comparison with control

**Fig. 4:**
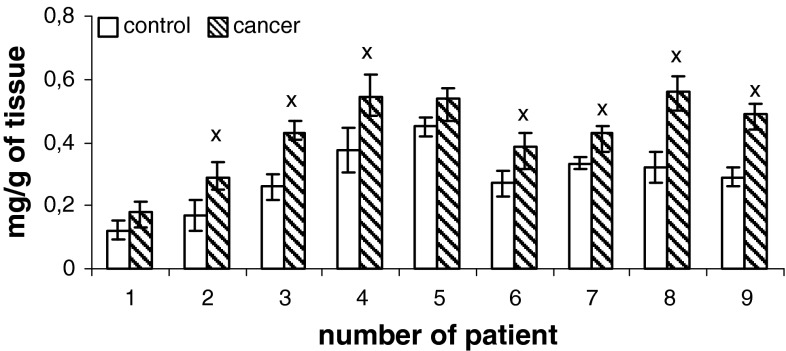
Content of phosphatidylcholine of cell membranes in human renal cancer of pT1 stage, G2 grade, without metastasis. Statistically significant differences for *p* < 0.05. ^x^ In comparison with control

Content of phospholipids (PtdIns, PtdSer, PtdEtn, PtdCho) of human renal cell membrane unmodified and of the membrane modified by neoplasm lesion are presented in Figs. 1, 2, 3, and 4. Generally, an increase in the content of all phospholipids is observed in the patients at pT1 stage, G2 grade, without metastasis (N0) with respect to the unaffected cells. However, phosphatidylcholine is most of all than other phospholipids, both in control tissue and in cancer tissues.

**Fig. 5:**
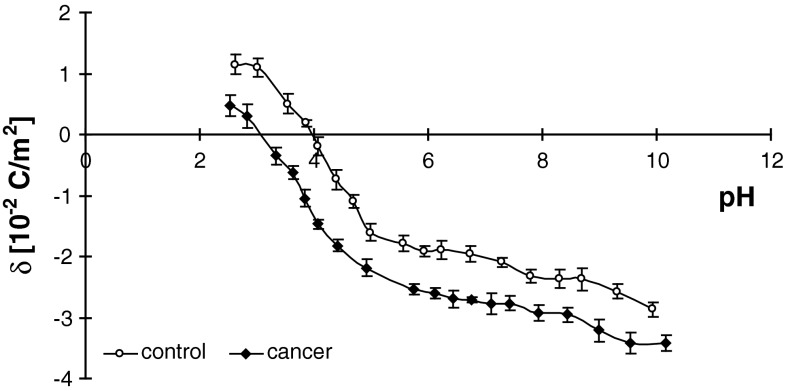
Dependence on pH of surface charge density of normal and renal cancer cells from several patients

Surface charge density dependence on pH of normal and renal cancer cell membrane are similarly shaped (Fig. 5). For example, an increase in positive surface charge density is observed at low pH values until a plateau is reached. Conversely, at high pH values, the proportion of negative charges present increases until it reaches a plateau. An increase in negative charge at low pH values and a decrease in positive charge at high pH is observed in human renal cancer cells compared to unaffected cells.

The surface fraction occupied by phospholipids as well as the surface concentration of acidic groups (*C*_TA_) and the surface concentration of basic groups (*C*_TB_) of human renal cell membrane unmodified and of the membrane modified by neoplasm lesion are presented in Table 1. The *C*_TA_ and *K*_BOH_ values of cell membranes modified by cancer transformation were higher than in unmodified cells, while *C*_TB_ and *K*_AH_ decreased in comparison (Table 1).

**Table 1:** *C*_TA_, *C*_TB_, *K*_AH_ and *K*_BOH_ of human renal cancer of pT1 stage, G2 grade, without metastasis

Patient no.	Patient status	*C*_TA_ (10^−7^ mol/m^2^)	*C*_TB_ (10^−7^ mol/m^2^)	*K*_AH_ (m^3^/mol)	*K*_BOH_ (10^7^ m^3^/mol)
1	Control	2.49 ± 0.11	1.23 ± 0.08	76.80 ± 0.90	1.68 ± 0.08
	Cancer	3.03 ± 0.10*	0.97 ± 0.09*	47.81 ± 1.10*	1.97 ± 0.12*
2	Control	2.67 ± 0.11	1.64 ± 0.08	51.08 ± 0.90	2.28 ± 0.10
	Cancer	2.96 ± 0.10*	0.77 ± 0.09*	30.71 ± 1.10*	2.59 ± 0.14*
3	Control	1.79 ± 0.11*	0.85 ± 0.09*	55.29 ± 1.08*	2.17 ± 0.11*
	Cancer	2.15 ± 0.12*	0.53 ± 0.06*	45.48 ± 1.18*	3.64 ± 0.12*
4	Control	1.87 ± 0.07*	0.95 ± 0.09*	76.13 ± 1.17*	1.20 ± 0.05*
	Cancer	2.12 ± 0.10*	0.62 ± 0.06*	56.48 ± 1.05*	2.26 ± 0.09*
5	Control	2.36 ± 0.09	0.69 ± 0.08	30.21 ± 0.90	1.93 ± 0.06
	Cancer	2.12 ± 0.10*	0.42 ± 0.09*	24.36 ± 1.10*	2.11 ± 0.09*
6	Control	2.49 ± 0.09	0.59 ± 0.07	41.26 ± 1.29	2.89 ± 0.08
	Cancer	2.64 ± 0.11*	0.36 ± 0.05*	32.57 ± 1.17*	3.21 ± 0.10*
7	Control	2.67 ± 0.12	0.56 ± 0.06	29.12 ± 0.99	1.65 ± 0.08
	Cancer	3.31 ± 0.14*	0.34 ± 0.05*	26.32 ± 1.01*	1.89 ± 0.10*
8	Control	2.54 ± 0.14	0.66 ± 0.07	30.24 ± 1.09	1.89 ± 0.07
	Cancer	3.53 ± 0.16*	0.48 ± 0.06*	27.14 ± 1.05*	2.07 ± 0.11*
9	Control	1.97 ± 0.07	0.93 ± 0.08	66.12 ± 1.16	1.22 ± 0.05
	Cancer	2.21 ± 0.10*	0.60 ± 0.05*	45.14 ± 1.01*	1.96 ± 0.09*

* Statistically significant difference (*p* < 0.05) compared to control

## Discussion

Tumorigenesis is associated with altered cellular behaviour and disorganization of membrane. The perturbations are reflected by changes in the content phospholipids and proteins and biological membranes. The phospholipids are an integral part of the membrane and determine its structure. The results presented in this work demonstrate that cancer changes provoke an increase in the amount of all phospholipids in cell membranes of renal. The data in the literature indicate that the levels of phospholipids were significantly higher in cancer renal as compared to normal renal (Prasad et al. 2005). The change in phospholipids composition of the membrane is a dependent process related to functional disorder of the cell. Phospholipid modifications cause membrane fluidity and affect many functions of the cell. Thus, alteration in the chemical composition and physical state of membrane lipids can modulate receptor, enzyme and transporter activity (Podo 1999).

Additional, higher amount of phospholipids can be due to enhanced cell membrane synthesis related to accelerated neoplasm cell replication (Ruiz-Cabello and Cohen 1992). The mechanisms that are responsible for an increase in the amount of phospholipids can vary depending on cell nature, cell growth phase and its malignancy. The greatest changes in the content of phosphatidylcholine and PtdEtn were observed in the first phase G_1_ of cell cycle, in which activity of the enzymes controlling biosynthesis, catabolism and metabolism of phospholipids attains maximum (Lunt and vander Heiden 2011). Our data show that PtdChol and PtdEtn are the main lipid classes in healthy human kidney and cancerous tissue (Figs. 1–4). Earlier reports are confirmed by these observations (Hoffmann et al. 2005).

Enhanced amount of phospholipids results in a higher amount of functional groups: amino, carboxy and phosphate groups. In acid medium (low pH), the charge of phospholipids is mainly due to amino groups whereas in basic medium (high pH) it is due to carboxy and phosphate groups. Increased amount of phospholipids can increase surface concentration function groups of kidney cell membrane. The main component of the kidney cell membrane outer layer is PtdChol and its higher content can provoke an increase in surface concentration function groups. The cancer transformation makes the association constant of negatively charged groups (*K*_AH_) lower and the association constant of positively charged ones (*K*_BOH_) higher (Table 1).

Hypoxia is one of the hallmarks of cancer. The presence of hypoxia has been demonstrated in different types of solid tumors. Intratumoral hypoxia is caused by the lack of functional blood vessels in proliferating tumors tissue, resulting in low intratumoral oxygen concentrations. If hypoxia is severe or prolonged cell death occurs (Turner et al. 2002; Samenza 2003). Hypoxia/reoxygenation and acidity-induced exposure of anionic phospholipids, most likely PtdSer and PtdEtn. Anionic phospholipids on tumor vessels could potentially provide markers for tumor vessel targeting and imaging (Ran et al. 2002; Ran and Thorpe 2002; Zhao et al. 1998). It seems that the alterations in the distribution of PtdSer, which is a component of the skeleton, could cause an increase in *C*_TA_ value (Table 1).

Beside the phospholipids that have been discussed in this work, the cell membrane charge is also affected by sialic acid being the component of glycolipids and glycoproteins. It has been supposed that sialic acid also influences surface concentration of acid and basic groups as well as association constants of positive and negative groups during cancer transformation. The sialic acid level of cancer cells in different tissues was increased with cancer progression (Nicol and Prasad 2002). Increased sialic acid content can provoke increased surface concentration of acid groups. This has been confirmed by the results of this work (Table 1).

The finding of a difference in physicochemical properties of renal cancer compared to normal renal tissue is potentially clinically useful. In other organs neoplastic tissue has been reported to have significantly different electrical properties compared with normal tissues (Inagaki et al. 2004). Such changes, if present in renal tissues, may be used to help localize cancer for biopsy and/or treatment, or to potentially aid in improving our diagnostic accuracy in the future.
